# Bifidobacterium longum BB536 is associated with improvements in gastrointestinal symptoms and odor-related metabolites in microbiota-defined subgroups of male athletes consuming a high-protein diet: exploratory randomized double‑blind placebo‑controlled trial

**DOI:** 10.1080/15502783.2026.2664664

**Published:** 2026-04-27

**Authors:** Shu Miyamoto, Shin Yoshimoto, Noriko Katsumata, Natsumi Mutoh, Noriyuki Iwabuchi, Toshitaka Odamaki, Daisuke Asaoka, Shuichi Machida

**Affiliations:** a Biotics Research Institute, Morinaga Milk Industry Co., Ltd., Zama-City, Kanagawa, Japan; b Department of Gastroenterology, Juntendo Tokyo Koto Geriatric Medical Center, Tokyo, Japan; c Graduate School of Health and Sports Science, Juntendo University, Inzai, Chiba, Japan

**Keywords:** Athletes, high-protein diet, diarrhea-related symptoms, body odor, microbiome stratification

## Abstract

**Background:**

High‑protein diets are widely used by athletes but may disturb the gut environment and increase production of odor‑related metabolites. Probiotic supplementation has been proposed as a strategy to support gastrointestinal function under such dietary stress. This study aimed to explore the effects of *Bifidobacterium longum* BB536 on gastrointestinal symptoms, gut microbiota, and odor‑related metabolites in male athletes consuming a high‑protein diet.

**Methods:**

In an exploratory, randomized, double‑blind, placebo‑controlled trial, 60 healthy male athletes (mean age: 18.62 ± 0.75 years; mean BMI: 22.35 ± 1.80 kg/m^2^) consumed a whey protein supplement (70 g/day) together with either BB536 (46 billion CFU/day, as measured at the start of the intervention) or placebo for 4 weeks. Gastrointestinal symptoms, gut microbiota composition, skin‑emitted volatile compounds, and fecal metabolites were assessed. Subgroup analyses based on responder status and baseline enterotype were conducted post hoc to generate hypotheses regarding microbiota‑dependent responses.

**Results:**

In the overall cohort, no significant between‑group differences were observed across gastrointestinal outcomes, gut microbiota indices, or metabolite profiles. Within the BB536 group, diarrhea‑related scores improved from baseline. Post hoc analyses suggested that increases in *Faecalibacterium* were evident among responders. Enterotype‑based patterns also emerged: individuals with *Ruminococcus*‑dominant microbiota showed higher skin‑emitted short‑chain fatty acids after BB536 intake, whereas those with *Faecalibacterium*‑dominant microbiota exhibited reductions in odor‑related metabolites, including methyl mercaptan and ammonia. Corresponding fecal metabolite shifts were modest.

**Conclusion:**

BB536 supplementation was associated with improvements in diarrhea‑related symptoms and odor‑related metabolites in specific microbiota‑defined subgroups. As these findings did not extend to the full cohort, they should be interpreted as exploratory and hypothesis‑generating. Baseline gut microbiota composition may influence probiotic responsiveness, warranting confirmatory trials with prespecified endpoints.

## 1. Introduction

The human gut harbors a diverse community of microorganisms that form a complex ecosystem essential for host health [1]. The gut microbiota contribute to intestinal barrier integrity [2], regulation of energy metabolism [3], and immune modulation [4]. Probiotics, defined as live microorganisms that confer health benefits when administered in adequate amounts [5], are widely recognized for their ability to stabilize the gut microbiota, support intestinal homeostasis, and ameliorate gastrointestinal symptoms such as diarrhea across a broad age range from infant to adults [6–9].

Recently, increasing attention has been directed toward the relationship between athletic performance and the gut environment. The composition and activity of the gut microbiota have been implicated in endurance capacity, recovery, and overall quality of life (QOL) in athletes. However, a considerable proportion of athletes suffer from gastrointestinal symptoms such as diarrhea during training or competition [10–12], which are often attributed to exercise-induced intestinal hypoperfusion and disruption of mucosal homeostasis [13,14]. Such symptoms are not only distressing but may directly impair performance. Probiotic supplementation has therefore been reported to attenuate performance decline following intense resistance exercise [15] and reduced incidence of respiratory illness in elite athletes [16].

Another important yet underexplored factor in this context is diet. Many athletes consume high-protein diets to support muscle repair and recovery [17]. However, excessive protein intake alters the gut microbiota composition and increase proteolytic fermentation, leading to the accumulation of putrefactive metabolites [18]. These changes have been associated with abdominal discomfort and pain [19], raising concerns that high-protein diets may predispose athletes to gut dysbiosis and performance decline. Despite the high prevalence of both protein supplementation and gastrointestinal complaints in athletes, few studies have directly examined the interaction between a high-protein diet and probiotic supplementation.

*Bifidobacterium longum* BB536 is a well-characterized probiotic strain with established safely and efficacy. It has been reported to regulate bowel movements, improve gastrointestinal symptoms, and benefit both constipation- and diarrhea-predominant conditions [20–22]. These properties suggest that BB536 may be particularly relevant for athletes at risk of gut disturbance due to high-protein diets and intensive training.

Beyond gastrointestinal health, athlete QOL is also shaped by factors such as body odor. Volatile metabolites such as ammonia, produced in the gut and transported into systemic circulation, can be released through the skin and contribute to odor generation [23–25]. Body odor may negatively influence athletes’ self-confidence, social interactions, and perceived well-being, particularly in competitive and team-based environments. To our knowledge, no prior intervention study has investigated whether probiotic supplementation can modulate skin-emitted metabolites associated with body odor in athletes.

In this randomized, double-blind, placebo-controlled trial, we evaluated the effects of *Bifidobacterium longum* BB536 supplementation in healthy male athletes consuming a high-protein diet. We assessed not only gastrointestinal symptoms and gut microbiota composition but also metabolite profiles in both feces and skin gas. By integrating these outcomes, this study addresses the unique intersection of gut environment, high-protein dietary stress, and athletic health/QOL, thereby providing novel insights into precision nutritional strategies for athletes.

## 2. Methods

### 2.1. Study population

Male students belonging to athletic clubs were recruited from the School of Health and Sports Science at Juntendo University between July and November 2024. All participants engaged in regular physical activity as part of their daily routines. Written informed consent was obtained from all participants prior to enrollment. A total of 60 participants were enrolled, based on the maximum feasible number of participants at the study sites.

The inclusion criteria were: males aged ≥18 years, in good health, and willing to consume the prescribed protein supplements. The exclusion criteria were: presence of severe medical conditions; regular use of medications that affect gastrointestinal function; lactose intolerance; high habitual protein intake (≥3.0 g/kg/day); severe food allergies; recent participation in other trials; and any other condition deemed inappropriate by the principal investigator. Eligibility was assessed by evaluating participants’ questionnaire responses and the contents of their dietary records.

### 2.2. Study design

This study was a randomized, double‑blind, placebo‑controlled, parallel‑group exploratory trial conducted at the Graduate School of Sport and Health Science, Juntendo University. Because the trial was prospectively positioned as exploratory, no primary endpoint was prespecified, and all outcomes—including gastrointestinal symptoms, microbiota indices, and metabolite profiles—were treated as exploratory and hypothesis‑generating.

The protocol adhered to the Declaration of Helsinki (2013 revision) and the Japanese Ethical Guidelines for Life Science and Medical Research Involving Human Subjects. Ethical approval was obtained from the Ethics Review Committee of the Graduate School of Sport and Health Science, Juntendo University (No. 2024-70, approval date: July 24, 2024), and the study was registered with UMIN (UMIN000055574).

Participants were stratified according to physical activity level (METs) and protein intake (g/kg/day), and then randomly assigned to groups using a computer-generated randomization list with block sizes of four. The allocation sequence was managed by an independent administrator who was not involved in participant allocation. The personnel responsible for assigning participants were blinded to the allocation sequence and had no access to it throughout the study.

Group allocation codes were concealed from both participants and researchers until all analyses were completed.

All study personnel—including investigators, data managers, and sponsor staff involved in study operations—remained fully blinded to group allocation throughout the trial. The randomization list and allocation codes were generated and securely maintained by the independent administrator, who was not involved in enrolment, data collection, data cleaning, or statistical analysis.

Prior to unblinding, the clinical database was formally locked on July 25, 2025, after all data queries had been resolved and data cleaning procedures were completed. Following the database lock, the predefined code‑breaking procedure was conducted, in which the independent administrator released the allocation codes solely to the designated statistician responsible for executing the prespecified analyses.

No other investigators or sponsor-employed personnel obtained access to allocation information until all prespecified analyses, conducted according to the Statistical Analysis Plan (SAP), had been completed.

### 2.3. Intervention

Participants were assigned to receive either *Bifidobacterium longum* BB536 (46 billion CFU/day, as measured at the start of the intervention) or placebo for 4 weeks. To standardize protein intake across participants, both groups consumed a commercially available whey protein supplement (DNS Whey Protein Supplement; Daiichi Sankyo Healthcare Co., Ltd.) at a daily dose of 70 g, providing 50 g protein. All test foods were provided by Morinaga Milk Industry Co., Ltd.

#### 2.3.1. Protein supplement composition and intake protocol

A 70g daily dose of whey protein supplement contains protein, carbohydrates (including lactose), fat, and standard flavors, emulsifiers, and sweeteners (Table S1). To minimize the potential influence of lactose intolerance on gastrointestinal outcomes, participants underwent screening using a structured questionnaire and medical history assessment prior to enrollment.

Participants were instructed to dissolve the protein supplement powder in 100–200 mL of water and to consume it after daily training sessions or at any timing on non-training days. Intake was performed twice per day throughout the intervention period.

#### 2.3.2. Probiotic and placebo capsules

Participants in the BB536 group received capsules providing 46 billion CFU/day, as measured at the start of the intervention.

Participants consumed the probiotic or placebo at any timing in a day. The placebo capsules matched the active capsules in appearance and taste and were composed of non-fermentable excipients without prebiotic properties (including starch, hydroxypropyl methylcellulose, gelling agent and caramel coloring), thereby minimizing any potential effects on gut microbial fermentation.

#### 2.3.3. Compliance monitoring

Participants recorded the intake of probiotic/placebo capsules, protein supplement consumption, bowel movements, stool consistency, and general physical condition in daily study diaries. Total weekly physical activity, including both scheduled club activities and additional self-initiated exercise, was documented and calculated as MET·h/week.

### 2.4. Dietary records

Dietary intake was recorded using the Calomeal app (Life Log Technology, Inc) [26] and daily study diaries during two separate periods: the week prior to the intervention, and the final week of the intervention. Regarding protein intake, protein intake from habitual dietary sources was calculated using the Calomeal application, while protein intake from supplementation was determined based on daily study diaries.

### 2.5. Microbiome analysis

Bacterial DNA extraction and PCR amplification were performed according to established protocols [27]. The V3-V4 hypervariable regions of the 16S rRNA gene were amplified using region-specific primers and sequenced with paired-end approach on the Illumina NextSeq 1000 platform, employing the NextSeq 1000/2000 P1 reagent kit (600 cycles) (Illumina, Inc., San Diego, CA, USA).

Resulting sequence data were processed and analyzed with QIIME2 (version 2022.8) [28]. After demultiplexing, sequences underwent quality filtering, denoising, merging, chimera detection and removal, and amplicon sequence variant (ASV) inference using DADA2 [29]. Taxonomic annotation of ASVs was performed by referencing the Greengenes2 database (version 2022.10), and alpha diversity indices were calculated in QIIME2.

For beta diversity and enterotype analysis, principal coordinate analysis (PCoA) and partitioning around medoid (PAM) clustering were conducted using R (version 4.3.2; R Foundation for Statistical Computing, Vienna, Austria). Enterotype clustering was performed at the genus level. utilizing Jensen–Shannon distance (JSD) and PAM clustering; the optimal number of clusters (enterotypes) was determined according to the Calinski–Harabasz index [30].

### 2.6. Metabolite analysis

All chemicals used for target analysis were purchased from FUJIFILM Wako Pure Chemical Corporation (Osaka, Japan), while the mobile phase and pH buffering solution were obtained from SHIMADZU GLC Ltd. (Kyoto, Japan) (Table S2).

The supernatant was extracted from fecal samples collected from participants as described previously [31]. HPLC analysis was conducted using the Nexera platform (SHIMADZU GLC Co., Ltd., Kyoto, Japan) equipped with two LC-40D pumps, a DGU-405 degasser, a SIL-40C autosampler, a CTO-40C column oven, a CBM-40 control module, and a CDD-10A VP conductivity detector. Metabolite separation was achieved using tandem ion-exclusion column (Shim-pack SCR-102H, 300 mm × 8.0 mm, 7 µm; SHIMADZU GLC Co., Ltd., Kyoto, Japan). The mobile phase and pH buffer were delivered at a flow rate of 0.8 mL/min, with the column temperature maintained at 50 °C, and injection volume set to 10 µL.

### 2.7. Bowel movement and stool consistency

Participants recorded their bowel movement frequency and stool consistency daily using standardized diaries. Stool consistency was self-assessed using the Bristol Stool Scale [32].

### 2.8. Questionnaires

The Izumo Scale, a validated questionnaire for assessing the quality of life (QOL) related to gastrointestinal symptoms in Japanese populations, was administered as a self-administered survey. It includes five dimensions: heartburn, stomach pain, dyspepsia, constipation, and diarrhea. Each dimension is scored from 0 (no symptoms) to 15 (severe symptoms) [33].

### 2.9. Skin gas analysis

Skin gas sampling was performed in the early morning prior to food intake, using a passive flux sampler (PFS) affixed to the participant's forearm for 1 hour. The analysis was conducted according to the method described previously [34,35]. Briefly, skin gas collected using the PFS was extracted and analyzed by gas chromatography-mass spectrometry (GC/MS), following component extraction procedures as previously reported. The quantification of emission levels was conducted by Human Metabolome Technologies, Inc (Yamagata, Japan).

### 2.10. Safety assessment

Adverse events were defined and assessed based on the “Common Terminology Criteria for Adverse Events (CTCAE) Version 5.0, Japanese translation (JCOG version).” Data collection was conducted using participant-maintained daily diaries.

### 2.11. Analysis populations

Because this was an exploratory randomized trial, analysis populations were defined to maximize transparency.

- The Full Analysis Set (FAS)—equivalent to a modified intention‑to‑treat population—included all randomized participants who received at least one dose of the assigned intervention. Participants without post‑baseline measurements were retained in the FAS for reporting but excluded from outcome‑specific analyses when no evaluable data were available.
- The Per‑Protocol Set (PP) included participants who completed the 4‑week intervention without major protocol deviations.

No imputation was performed for missing data. Unless otherwise specified, exploratory analyses (including responder‑ and enterotype‑based analyses) were conducted within the FAS.

### 2.12. Statistical analysis

Because all outcomes were exploratory, analyses were hypothesis‑generating and not powered for confirmatory inference. Microbiota diversity and related outcomes were assessed at baseline (week 0) and after the intervention (week 4) as follows: Alpha diversity was compared between groups using the Mann–Whitney U test. Beta diversity was assessed with permutational multivariate analysis of variance (PERMANOVA). Gut microbial relative abundance between groups was compared following centered log-ratio (CLR) transformation, employing Quade’s nonparametric analysis of covariance (ANCOVA). Within-group comparisons were performed using the ALDEx2 package. Gut metabolites and skin gas were compared between groups using Quade’s nonparametric ANCOVA, and within-group comparisons were conducted using the Wilcoxon signed-rank test.

Background information, dietary records, and bowel movement, and stool consistency were analyzed using the Student’s t-test for between-group comparisons and the paired t-test for within-group comparisons. Izumo Scale scores were analyzed for between-group differences using the Mann–Whitney U test based on the change from baseline to week 4, and within-group comparisons were conducted using the Wilcoxon signed-rank test.

All statistical analyses were performed using R (v4.3.2; R Foundation for Statistical Computing, Vienna, Austria) and SPSS (v30.0; IBM Corp., Armonk, NY, USA), and no imputation was applied for missing data. For microbiome analyses, multiple testing corrections were applied to microbial relative abundance comparisons using the Benjamini–Hochberg false discovery rate (FDR) method. Adjusted q-values < 0.05 were considered statistically significant, and q-values < 0.10 were interpreted as indicative of trends. Other outcomes (e.g. metabolite concentrations and skin gas profiles) were analyzed without FDR adjustment because these were predefined endpoints with a limited number of comparisons.

All analyses performed after unblinding followed the prespecified Statistical Analysis Plan (SAP). The SAP was finalized on July 18, 2025.

Exploratory post‑hoc analyses conducted by sponsor‑employed statisticians after unblinding were independently reviewed by academic investigators and were not used to modify or influence the prespecified analysis results or their interpretation.

## 3. Result

Consistent with the exploratory nature of this trial, no statistically significant between‑group differences were observed for any of the prospectively evaluated outcomes in the full analysis population. Although diarrhea‑related Izumo scores improved within the BB536 group, this change did not differ significantly from the placebo group. Therefore, subsequent responder‑ and enterotype‑stratified analyses were treated as post‑hoc exploratory analyses to generate hypotheses rather than to indicate confirmatory treatment effects.

### 3.1. Participant background

**Figure 1.**
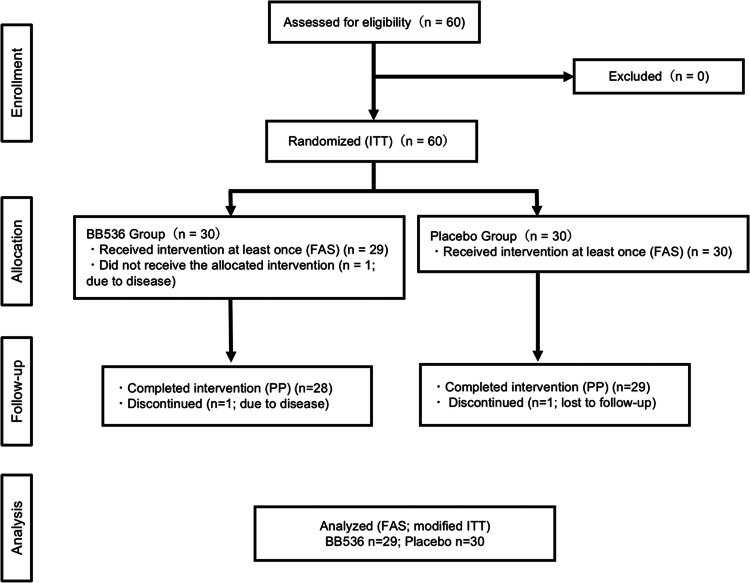
CONSORT flow diagram showing participant flow through the study. Analyzed refers to FAS (modified ITT): Placebo *n* = 30; BB536 *n* = 29. ITT = all randomized (*n* = 60); PP = completed per protocol (Placebo *n* = 29; BB536 *n* = 28). Participants without post‑baseline (post‑dose) data were included in the FAS for transparency but were excluded from outcome analyses requiring such data; no imputation was applied.

Across the full analysis population (FAS), no significant between‑group differences were observed in baseline characteristics. The CONSORT flow diagram is shown in Figure 1. Of the 60 participants who were randomized, 57 completed the assigned intervention (Placebo: *n* = 29; BB536: *n* = 28). In the placebo group, 29 of 30 participants (97%) completed the intervention as per protocol, while one participant (3%) was lost to follow-up during the intervention period and therefore only partially completed the intervention. In the BB536 group, 28 of 30 participants (93%) completed the intervention as planned. One participant (3%) discontinued the intervention due to disease and was thus classified as having partially completed the intervention, and another participant (3%) developed a disease prior to the initiation of the intervention and did not receive any intervention. Accordingly, the Full Analysis Set (FAS; modified ITT) included all participants who received at least one dose of the assigned intervention (Placebo: *n* = 30; BB536: *n* = 29). As described in Methods, participants without post‑baseline measurements were excluded only from analyses for which no evaluable data were available. All adverse events showed no association with the intervention of the study foods.

Baseline characteristics, including age and BMI, did not differ between groups (Table 1). Detailed dietary data are shown in Supplementary Table S3, which revealed that protein supplementation significantly increased the participants' total protein intake per body weight (g/kg/day) at week 4 in both groups (placebo group: *p* < 0.001, BB536 group: *p* < 0.001). In the BB536 group, significant increases in calorie, protein (habitual dietary sources), and fat intake were observed at week 4, but no significant differences were observed between groups. Additionally, no significant differences were observed between or within groups in dietary fiber and carbohydrate intake. No significant within-group or between-group differences were observed for training loads levels (MET·h/week) during the study period (Table S3, S4).

**Table 1.** Participant background.

	Placebo (*n* = 30)	BB536 (*n* = 29)	*p* value
Age (year)	18.60 ± 0.67	18.66 ± 0.86	0.785
Height (cm)	174.73 ± 6.33	173.19 ± 6.46	0.360
Weight (kg)	68.33 ± 6.32	67.13 ± 7.58	0.512
BMI (kg/m^2^)	22.37 ± 1.55	22.37 ± 2.10	1.000

Values (means ± SD) were compared between groups. Statistical significance is indicated as follows: Student's t-test.

### 3.2. Abdominal symptoms

Across the full analysis population, no significant between‑group differences were observed for gastrointestinal outcomes (Table S5). However, gastrointestinal QOL assessed by the Izumo scale demonstrated a significant within-group reduction in diarrhea-related scores in the BB536 group at week 4 compared with baseline (*p* = 0.044) (Table 2). This within‑group improvement did not differ significantly from the placebo group.

**Table 2.** The scores of Izumo scale.

	Placebo			BB536		
	week 0 (*n* = 30)	week 4 (*n* = 29)	*p* value	week 0 (*n* = 29)	week 4 (*n* = 28)	*p* value
Heartburn	0.76 ± 1.52	0.38 ± 1.13	0.058#	0.32 ± 0.71	0.29 ± 0.84	0.863
Stomach pain	0.34 ± 0.84	0.21 ± 0.92	0.498	0.43 ± 1.24	0.29 ± 0.92	0.438
Dyspepsia	0.48 ± 0.77	0.48 ± 1.38	0.341	0.54 ± 1.50	0.50 ± 1.30	1.000
Constipation	1.07 ± 1.51	0.93 ± 2.41	0.467	1.11 ± 2.73	0.96 ± 2.40	0.763
Diarrhea	1.31 ± 1.80	1.55 ± 2.55	0.774	1.32 ± 2.41	0.61 ± 1.57	0.044*

Values (means ± SD) were compared within groups. Statistical significance is indicated as follows: ^#^*p* < 0.1; *p < 0.05 with Wilcoxon signed-rank test.

### 3.3. Gut microbiota composition

In the full analysis population, no significant between‑group differences were observed in alpha diversity, beta diversity, or taxonomic composition.

All subgroup findings (responder/enterotype) were post‑hoc, underpowered, and are reported solely as hypothesis‑generating.

**Figure 2.**
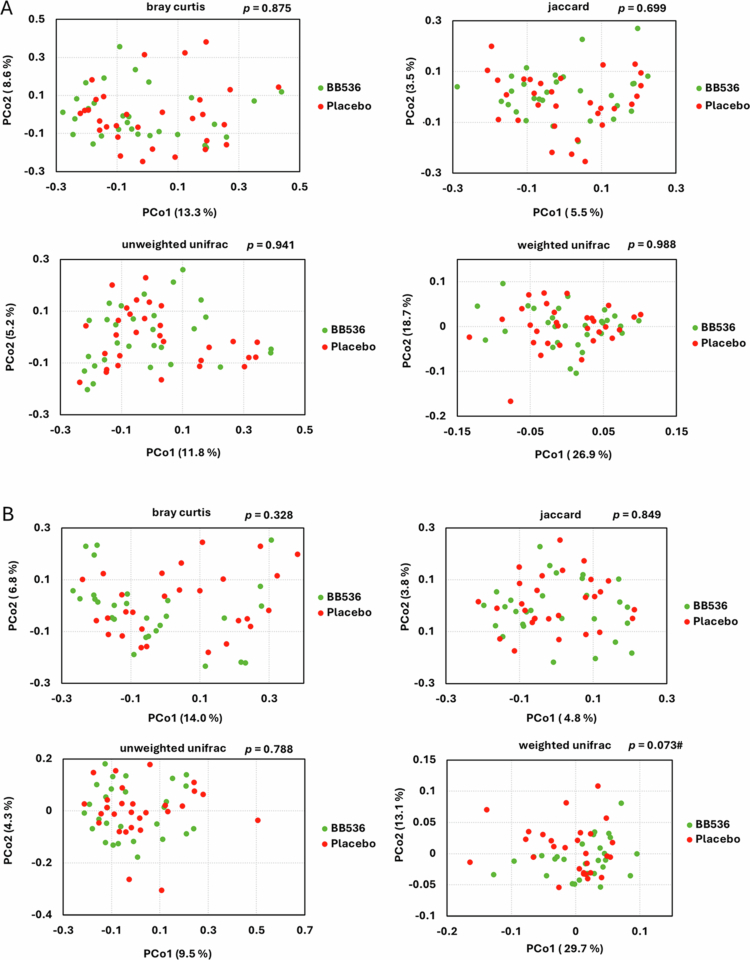
Indices Index of *β* diversity on baseline and week 4. Principal coordinate analysis (PCoA) as seen through the first two principal coordinates (PCo1 and PCo2) based on Jensen–Shannon distance (JSD) calculated by the gut microbiota composition at the ASV level at baseline (A) and week 4 (B). Red: Placebo, Green: BB536 Statistical significance is indicated as follows: ^#^*p* < 0.1 with PERMANOVA Red: Placebo, Green: BB536.

There were no significant between‑group in alpha diversity indices (Table S6). On the contrary, beta diversity analysis showed a trend toward separation between groups at week 4 (Weighted UniFrac, *p* = 0.073) (Figure 2). At the phylum and genus levels, no major group‑level shifts were observed (Table 3).

**Table 3.** Composition of gut microbial relative abundance in Placebo and BB536 groups.

	week 0		week 4				
Phylum	Placebo (*n* = 30)	BB536 (*n* = 29)	Placebo (*n* = 29)	BB536 (*n* = 27)	Between groups *q* value	Within-group *q* value	
						Placebo	BB536
*Firmicutes_A*	0.654 ± 0.117	0.663 ± 0.132	0.647 ± 0.127	0.722 ± 0.090	0.103	0.585	0.189
*Actinobacteriota*	0.138 ± 0.098	0.131 ± 0.114	0.120 ± 0.082	0.081 ± 0.053	0.809	0.618	0.573
*Firmicutes_D*	0.074 ± 0.093	0.064 ± 0.059	0.063 ± 0.051	0.063 ± 0.062	0.809	0.876	0.654
*Bacteroidota*	0.086 ± 0.064	0.081 ± 0.057	0.122 ± 0.070	0.091 ± 0.067	0.565	0.116	0.324
*Firmicutes_C*	0.032 ± 0.041	0.044 ± 0.046	0.029 ± 0.030	0.034 ± 0.039	0.862	0.668	0.728
*Unclassified bacteria*	0.008 ± 0.014	0.009 ± 0.016	0.008 ± 0.009	0.004 ± 0.007	0.210	0.468	0.522
*Proteobacteria*	0.005 ± 0.006	0.006 ± 0.008	0.006 ± 0.010	0.003 ± 0.004	0.809	0.879	0.724

	week 0		week 4				
Genus	Placebo (*n* = 30)	BB536 (*n* = 29)	Placebo (*n* = 29)	BB536 (*n* = 27)	Between groups *q* value	Within-group *q* value	
						Placebo	BB536
*Blautia_A_141781*	0.165 ± 0.080	0.170 ± 0.083	0.162 ± 0.077	0.191 ± 0.075	0.645	1	1
*Ruminococcus_B*	0.056 ± 0.071	0.045 ± 0.087	0.052 ± 0.060	0.041 ± 0.055	0.891	1	1
*Bifidobacterium_388775*	0.132 ± 0.098	0.123 ± 0.113	0.113 ± 0.080	0.075 ± 0.051	0.645	1	1
*Fusicatenibacter*	0.034 ± 0.031	0.037 ± 0.030	0.027 ± 0.025	0.050 ± 0.042	0.269	1	1
*Agathobacter_164117*	0.048 ± 0.048	0.035 ± 0.042	0.048 ± 0.064	0.038 ± 0.052	0.891	1	1
*Faecalibacterium*	0.083 ± 0.061	0.083 ± 0.064	0.078 ± 0.046	0.101 ± 0.069	0.645	1	1
*Anaerostipes*	0.038 ± 0.041	0.039 ± 0.042	0.038 ± 0.045	0.044 ± 0.053	0.645	1	1
*Gemmiger_A_73129*	0.032 ± 0.024	0.027 ± 0.028	0.030 ± 0.023	0.027 ± 0.026	0.891	1	1
*Streptococcus*	0.039 ± 0.061	0.031 ± 0.037	0.031 ± 0.042	0.030 ± 0.039	0.734	1	1
*Mediterraneibacter_A_155507*	0.024 ± 0.043	0.028 ± 0.030	0.025 ± 0.035	0.035 ± 0.044	0.645	1	1
*Phocaeicola_A_858004*	0.037 ± 0.034	0.037 ± 0.032	0.058 ± 0.053	0.034 ± 0.023	0.645	1	1
*Anaerobutyricum*	0.023 ± 0.025	0.027 ± 0.023	0.027 ± 0.023	0.026 ± 0.020	0.891	1	1
*Dorea_A*	0.013 ± 0.021	0.018 ± 0.016	0.014 ± 0.020	0.023 ± 0.020	0.269	1	1
*Dialister*	0.007 ± 0.014	0.011 ± 0.023	0.010 ± 0.021	0.009 ± 0.014	0.733	1	1
*Ruminococcus_E*	0.016 ± 0.027	0.020 ± 0.034	0.018 ± 0.046	0.019 ± 0.035	0.891	1	1
*Unclassified Lachnospiraceae*	0.015 ± 0.019	0.016 ± 0.020	0.014 ± 0.030	0.014 ± 0.017	0.891	1	1
*Bacteroides_H*	0.035 ± 0.038	0.031 ± 0.029	0.044 ± 0.037	0.040 ± 0.047	0.891	1	1
*Faecalimonas*	0.009 ± 0.013	0.006 ± 0.008	0.008 ± 0.012	0.006 ± 0.010	0.733	1	1
*Agathobaculum*	0.008 ± 0.006	0.009 ± 0.005	0.009 ± 0.006	0.009 ± 0.005	0.891	1	1
*Enterocloster*	0.008 ± 0.012	0.013 ± 0.011	0.010 ± 0.013	0.011 ± 0.011	0.733	1	1
*Parabacteroides_B_862066*	0.007 ± 0.008	0.008 ± 0.009	0.010 ± 0.011	0.011 ± 0.015	0.985	1	1

Data (phylum and genus) are presented as the means for taxa with a mean relative abundance greater than 0.5%. Values (means ± SD) were compared between groups and within groups. Statistical significance is indicated as follows: (between groups) Quade’s nonparametric ANCOVA and (within group) ALDEx2 package. False discovery rate (FDR) correction using the Benjamini–Hochberg method was applied to microbial relative abundance comparisons; adjusted *q*-values < 0.05 were considered significant and *q*-values < 0.10 were interpreted as trends.

Responder analysis among BB536 participants who showed improved diarrhea scores (responders, *n* = 9) demonstrated a significant increase in *Faecalibacterium* abundance after the intervention (*q* = 0.014). *Ruminococcus* and *Streptococcus* tended to be lower in responders than in non‑responders (*n* = 19) (*q* = 0.076 and *q* = 0.071, respectively) (Table 4).

**Table 4.** Composition of gut microbial relative abundance in Non-Responders and Responders.

	week 0		week 4				
	Non-Responders (*n* = 19)	Responders (*n* = 9)	Non-Responders (*n* = 19)	Responders (*n* = 9)	Between groups	Within-group *q* value	
Genus					*q* value	Non-Responders	Responders
*Blautia_A_141781*	0.172 ± 0.088	0.172 ± 0.079	0.186 ± 0.059	0.202 ± 0.106	0.951	1.000	1.000
*Ruminococcus_B*	0.041 ± 0.077	0.058 ± 0.114	0.051 ± 0.060	0.020 ± 0.036	0.076#	1.000	1.000
*Bifidobacterium_388775*	0.126 ± 0.134	0.121 ± 0.068	0.074 ± 0.055	0.076 ± 0.041	0.951	1.000	1.000
*Fusicatenibacter*	0.040 ± 0.033	0.033 ± 0.024	0.052 ± 0.049	0.044 ± 0.022	0.951	1.000	1.000
*Agathobacter_164117*	0.039 ± 0.041	0.013 ± 0.013	0.045 ± 0.060	0.023 ± 0.026	0.951	1.000	1.000
*Faecalibacterium*	0.080 ± 0.072	0.080 ± 0.043	0.075 ± 0.063	0.155 ± 0.048	0.014*	1.000	0.214
*Anaerostipes*	0.042 ± 0.049	0.033 ± 0.028	0.049 ± 0.062	0.035 ± 0.028	0.951	1.000	1.000
*Gemmiger_A_73129*	0.031 ± 0.032	0.023 ± 0.017	0.028 ± 0.030	0.023 ± 0.014	0.951	1.000	1.000
*Streptococcus*	0.032 ± 0.041	0.032 ± 0.032	0.037 ± 0.044	0.014 ± 0.016	0.071#	1.000	1.000
*Mediterraneibacter_A_155507*	0.034 ± 0.034	0.015 ± 0.017	0.043 ± 0.051	0.018 ± 0.017	0.951	1.000	1.000
*Phocaeicola_A_858004*	0.039 ± 0.035	0.031 ± 0.029	0.036 ± 0.023	0.030 ± 0.022	0.949	1.000	1.000
*Anaerobutyricum*	0.022 ± 0.022	0.037 ± 0.026	0.022 ± 0.016	0.036 ± 0.026	0.951	1.000	1.000
*Dorea_A*	0.019 ± 0.018	0.018 ± 0.014	0.023 ± 0.020	0.022 ± 0.020	0.951	1.000	1.000
*Dialister*	0.008 ± 0.017	0.017 ± 0.033	0.010 ± 0.016	0.008 ± 0.009	0.951	1.000	1.000
*Ruminococcus_E*	0.010 ± 0.030	0.036 ± 0.040	0.009 ± 0.026	0.040 ± 0.045	0.951	1.000	1.000
*Unclassified Lachnospiraceae*	0.019 ± 0.023	0.012 ± 0.012	0.014 ± 0.015	0.015 ± 0.021	0.951	1.000	1.000
*Bacteroides_H*	0.029 ± 0.029	0.029 ± 0.027	0.045 ± 0.055	0.030 ± 0.021	0.951	1.000	1.000
*Agathobaculum*	0.009 ± 0.009	0.010 ± 0.005	0.009 ± 0.006	0.008 ± 0.004	0.951	1.000	1.000
*Enterocloster*	0.014 ± 0.011	0.012 ± 0.012	0.012 ± 0.013	0.008 ± 0.005	0.951	0.998	1.000
*Parabacteroides_B_862066*	0.008 ± 0.005	0.008 ± 0.008	0.013 ± 0.017	0.007 ± 0.007	0.951	1.000	1.000

Values (means ± SD) were compared between groups and within groups. Statistical significance is indicated as follows: # *q* < 0.1; *q < 0.05 with (between groups) Quade’s nonparametric ANCOVA and (within group) ALDEx2 package. All q-values were obtained after false discovery rate (FDR) correction using the Benjamini–Hochberg method.

### 3.4. Skin gas profiles

Across the full analysis population, no significant between‑group differences were observed for skin‑emitted volatile metabolites.

Volatile metabolites can be released through the skin via the bloodstream. To evaluate whether BB536 affects these metabolites, we measured short-chain fatty acids (SCFAs) and ammonia in skin gas samples. Overall, no significant between‑group differences were observed. Within the BB536 group, propionic acid significantly increased (*p* = 0.019), whereas methyl mercaptan significantly decreased (*p* = 0.008) at week 4 (Table 5).

**Table 5.** Effects of BB536 intake on skin gas.

Metabolite (ng cm^−2^ h^−1^)	week 0		week 4				
	Placebo (*n* = 30)	BB536 (*n* = 29)	Placebo (*n* = 29)	BB536 (*n* = 28)	Between group	Within-group *p* value	
					*p* value	Placebo	BB536
Acetic acid	120.91 ± 77.45	152.47 ± 155.42	103.88 ± 38.63	114.01 ± 56.92	0.860	0.642	0.733
Propionic acid	5.16 ± 1.95	4.99 ± 1.33	5.77 ± 2.00	6.18 ± 2.11	0.532	0.265	0.019*
Butyric acid	0.30 ± 0.16	0.30 ± 0.16	0.35 ± 0.16	0.35 ± 0.18	0.767	0.243	0.425
Valeric acid	0.41 ± 0.36	0.42 ± 0.23	0.44 ± 0.24	0.47 ± 0.33	0.946	0.482	0.716
Isovaleric acid	3.33 ± 1.89	3.28 ± 1.28	3.66 ± 1.51	3.34 ± 1.30	0.537	0.256	0.909
Acetone	2.69 ± 0.85	2.72 ± 0.88	2.98 ± 1.27	2.78 ± 0.99	0.541	0.370	0.990
Diacetyl	0.19 ± 0.16	0.21 ± 0.17	0.19 ± 0.12	0.21 ± 0.18	0.852	0.634	0.785
Methyl mercaptan	1.00 ± 0.49	1.09 ± 0.55	0.78 ± 0.31	0.79 ± 0.29	0.792	0.103	0.008*
Ammonia	112.17 ± 180.71	132.78 ± 158.57	75.36 ± 109.29	124.32 ± 183.08	0.971	0.611	0.156

Values (means ± SD) were compared between groups and within groups. Statistical significance is indicated as follows: # *p* < 0.1; *p < 0.05 with (between groups) Quade’s nonparametric ANCOVA and (within group) Wilcoxon signed-rank test.

To explore whether changes in skin gas composition were associated with gut microbiota structure, participants were clustered according to their baseline gut microbiota composition at pre-intervention. Participants were classified into the following two enterotypes (Table S7).

Cluster 1 (*Ruminococcus*-dominant, *n* = 18): At week 4, the BB536 group showed a tendency toward higher acetic acid compared with the placebo group (*p* = 0.050). Within-group comparison revealed a trend toward increased propionic acid (*p* = 0.050) and a significant increase in butyric acid (*p* = 0.035), whereas the placebo group showed a trend toward decreased isovaleric acid (*p* = 0.066) and methyl mercaptan (*p* = 0.086) (Table 6).

**Table 6.** Effects of BB536 intake on skin gas for cluster 1 and 2.

Cluster 1							
Metabolite (ng cm^−2^ h^−1^)	week 0		week 4				
	Placebo (*n* = 10)	BB536 (*n* = 8)	Placebo (*n* = 9)	BB536 (*n* = 8)	Between groups	Within-group *p* value	
					*p* value	Placebo	BB536
Acetic acid	116.84 ± 99.80	99.96 ± 53.08	79.20 ± 38.75	130.33 ± 55.77	0.050#	0.953	0.161
Propionic acid	5.74 ± 2.48	5.14 ± 1.57	6.34 ± 2.50	7.95 ± 2.50	0.190	0.441	0.050#
Butyric acid	0.31 ± 0.19	0.27 ± 0.15	0.34 ± 0.16	0.40 ± 0.16	0.399	0.722	0.035*
Valeric acid	3.28 ± 1.64	3.38 ± 1.55	4.60 ± 1.65	4.22 ± 1.34	0.613	0.678	0.779
Isovaleric acid	0.38 ± 0.43	0.37 ± 0.30	0.33 ± 0.16	0.34 ± 0.12	0.662	0.066#	0.674
Acetone	2.91 ± 1.16	2.85 ± 1.12	0.95 ± 0.32	1.06 ± 0.38	0.189	0.374	1.000
Diacetyl	0.25 ± 0.25	0.20 ± 0.19	0.13 ± 0.04	0.19 ± 0.07	0.933	0.594	0.779
Methyl mercaptan	1.11 ± 0.53	1.28 ± 0.74	0.27 ± 0.09	0.17 ± 0.06	0.335	0.086#	0.161
Ammonia	199.88 ± 283.38	107.09 ± 109.89	144.90 ± 48.30	197.83 ± 69.94	0.319	0.260	0.889

Cluster 2							
Metabolite (ng cm^−2^ h^−1^)	week 0		week 4				
	Placebo (*n* = 20)	BB536 (*n* = 21)	Placebo (*n* = 20)	BB536 (*n* = 20)	Between group	Within-group *p* value	
					*p* value	Placebo	BB536
Acetic acid	122.62 ± 70.37	175.14 ± 181.10	114.98 ± 33.89	107.48 ± 57.46	0.202	0.681	0.263
Propionic acid	4.98 ± 1.69	5.02 ± 1.24	5.51 ± 1.74	5.46 ± 1.47	0.899	0.411	0.156
Butyric acid	0.30 ± 0.15	0.32 ± 0.17	0.36 ± 0.17	0.32 ± 0.18	0.361	0.225	0.794
Valeric acid	3.18 ± 1.92	3.23 ± 1.24	3.24 ± 1.26	2.98 ± 1.13	0.887	0.502	0.654
Isovaleric acid	0.44 ± 0.34	0.44 ± 0.21	0.49 ± 0.25	0.52 ± 0.38	0.718	0.852	0.601
Acetone	2.62 ± 0.71	2.72 ± 0.78	2.83 ± 1.39	2.82 ± 0.98	0.759	0.823	0.888
Diacetyl	0.17 ± 0.11	0.21 ± 0.17	0.19 ± 0.12	0.21 ± 0.18	0.786	0.380	0.867
Methyl mercaptan	0.94 ± 0.49	1.02 ± 0.47	0.80 ± 0.33	0.77 ± 0.32	0.824	0.538	0.028*
Ammonia	64.53 ± 92.68	147.51 ± 177.99	68.18 ± 92.66	113.79 ± 181.12	0.583	0.852	0.053#

Values (means ± SD) were compared between groups and within groups. Statistical significance is indicated as follows: # *p* < 0.1; *p < 0.05 with (between groups) Quade’s nonparametric ANCOVA and (within group) Wilcoxon signed-rank test.

Cluster 2 (*Faecalibacterium*-dominant, *n* = 41): No between-group differences were observed, but within the BB536 group, methyl mercaptan significantly decreased (*p* = 0.028) and ammonia tended to decrease (*p* = 0.053) at week 4 (Table 6).

### 3.5. Gut metabolites profiles

In the full analysis population, fecal metabolite concentrations showed no significant between‑group differences at week 4.

We evaluated whether changes in skin‑gas profiles corresponded to alterations in fecal metabolites. In the overall cohort, the BB536 group showed trends toward increased isobutyric acid (*p* = 0.077) and isovaleric acid (*p* = 0.072) (Table S8). As part of the exploratory post‑hoc analyses, cluster-based analysis revealed that in cluster 1, the BB536 group exhibited significantly higher isovaleric acid concentration than the placebo group at week 4 (*p* = 0.021). Within-group comparisons also showed a decrease in isovaleric acid in the placebo group (*p* = 0.043). No significant differences in fecal metabolites were detected in cluster 2 (Table S9).

## 4. Discussion

In this randomized, double-blind, placebo-controlled trial, we investigated the effects of *B. longum* BB536 supplementation in healthy male athletes consuming a high-protein diet. We assessed gastrointestinal symptoms, gut microbiota composition, and metabolites in both feces and skin gas. To our knowledge, this is the first intervention study to simultaneously evaluate the interaction between probiotic supplementation, high-protein dietary stress, and odor-related metabolites in athletes. Consistent with the study’s prospectively declared exploratory nature (no prespecified primary endpoint), findings are interpreted as hypothesis-generating rather than confirmatory, especially given that no significant between-group differences were observed in the full analysis population.

To clarify the operational definition of the “high‑protein diet” used in this study, we calculated total protein intake relative to body mass (g/kg/day). During the intervention period, participants in both groups consumed protein within the range commonly recognized in sports nutrition literature as constituting a high‑protein diet for athletes (approximately 1.4–2.0 g/kg/day), a range supported by meta‑analytic evidence [36]. Importantly, the increase in protein intake per kg of body weight did not differ between the placebo and BB536 groups, indicating that differences in protein consumption are unlikely to have confounded the observed results. Therefore, the effects reported in this study can be interpreted as occurring under conditions of equivalently increased protein intake between groups, consistent with contemporary definitions of a high‑protein dietary regimen in athletic populations.

Gastrointestinal symptoms such as diarrhea and abdominal bloating have been reported to directly impair athletic performance during competition [11,37], and 30–70% of endurance athletes are estimated to experience such symptoms [10,38]. In the present study, diarrhea-related scores improved significantly in the BB536 group, and responders exhibited increased abundance of *Faecalibacterium.* We have previously reported that BB536 intake increases *Faecalibacterium* levels in healthy adults [31,39]. *Faecalibacterium* is a butyrate producer that contributes to intestinal barrier integrity and suppression of inflammation [40]. A reduction in *Faecalibacterium prausnitzii* has been reported in patients with inflammatory bowel disease [41], while other probiotic studies have linked increased *Faecalibacterium* abundance with improvements in diarrhea-related symptoms [42]. Moreover, *Faecalibacterium prausnitzii* is enriched in athletes and may be associated with greater exercise capacity [43]. Although exercise performance was not assessed here, the observed increase in *Faecalibacterium* suggests a potential biomarker of probiotic responsiveness in athletes. As gastrointestinal symptoms are reported more frequently among female athletes [44,45], future trials should include this population.

Another notable finding was the effect of BB536 supplementation on skin-emitted metabolites, assessed as a non-invasive surrogate of systemic metabolism [46]. Within the BB536 group, propionic acid increased and methyl mercaptan decreased in skin gas. SCFAs such as acetate, propionate, butyrate have been associated with mitochondrial function, glucose metabolism, inflammation control, and muscle maintenance, all of which are relevant to performance [47]. Importantly, skin-emitted metabolites were treated as a non-invasive proxy rather than a systemic validated surrogate; because no blood metabolites were collected, mechanistic interpretations are hypothesis-generating and causal inferences cannot be drawn.

Cluster analysis revealed that participants with *Ruminococcu*s-dominant microbiota showed increases in SCFAs, while those with *Faecalibacterium*-dominant microbiota exhibited reductions in odor-related compounds including methyl mercaptan and ammonia. Dietary fiber and fermentable carbohydrates are important modulators of protein fermentation and related metabolites. In this study, dietary fiber and carbohydrate intake were assessed, and no significant differences were observed between or within groups during the intervention period. Therefore, the changes in gut microbiota and metabolites observed in this study are unlikely to be attributable to differences in fiber intake. Nevertheless, future research should examine the interaction between high-protein diets, probiotics, and dietary fiber in greater detail. *Ruminococcus* degrades dietary fiber and produces SCFAs [48], and co-culture with *Bifidobacterium* has been shown to enhance resistant starch utilization and acetate production [49]. These interactions may explain the cluster-specific SCFA increases observed in this study. However, despite these subgroup‑specific patterns, none of these findings were supported by significant between‑group differences in the full analysis population. The apparent effects of BB536 emerged only in small, post‑hoc defined responder and enterotype subgroups; accordingly, they should be regarded as exploratory signals requiring prospective validation.

Fecal SCFA concentrations were additionally measured to explore whether skin gas changes were reflected in the intestinal lumen, but no consistent between-group differences were detected. Fecal SCFAs may not accurately reflect systemic SCFA dynamics, as individual variation in absorption and utilization is considerable. This interpretation is consistent with findings by Nogal et al., who reported a poor correlation between intestinal and circulating SCFA concentrations [50]. Direct measurement of blood metabolites in future studies will be needed to clarify these relationships.

We also observed a significant decrease in methyl mercaptan in skin gas following BB536 intake. Body odor is an important factor for athlete quality of life, and negative psychological states after intense exercise or defeat may worsen its perception [51]. Notably, in the *Faecalibacterium-*dominant cluster, ammonia tended to decrease alongside methyl mercaptan. Ammonia is produced in the gut via urea hydrolysis and amino acid deamination [52] and can be emitted through the skin [24,25]. Butyrate-producing bacteria lower intestinal pH and suppress urease and deaminase activity [53], which may reduce ammonia production. SCFAs have also been proposed to improve nitrogen metabolism and enhance detoxification [54]. Furthermore, acetate produced by BB536 may support butyrate production by *Faecalibacterium* [55], thereby contributing to a reduction in odor-related metabolites. The potential clinical relevance of these findings relates to their influence on perceived body odor, which can affect self-confidence, social interactions, and overall quality of life (QOL) in athletes. Thus, reductions in odor‑related metabolites such as methyl mercaptan and ammonia may help improve social comfort and psychological well‑being, even though athletic performance outcomes were not directly measured in this study. Because these subgroup analyses were post hoc nature and underpowered, the results should be interpreted as preliminary. The mechanistic explanations proposed here are plausible but remain hypothetical in the absence of direct measurements of functional pathways, enzymatic activities, or omics-based host–microbe interactions. Future studies incorporating metagenomic, metatranscriptomic, and host biomarker analyses will be essential to confirm these mechanisms and establish their physiological significance.

In conclusion, BB536 supplementation was not associated with consistent improvements across the entire cohort. Although diarrhea‑related gastrointestinal symptoms improved within the BB536 group, and exploratory responder‑ and enterotype‑based analyses revealed increases in *Faecalibacterium* and reductions in odor‑related metabolites, these effects were limited to specific microbiota‑defined subgroups. This pattern suggests that probiotic responsiveness in athletes consuming a high‑protein diet may be highly individualized and dependent on baseline gut microbiota composition. Given that cohort‑level between‑group superiority was not demonstrated, the present findings should be interpreted as hypothesis‑generating rather than confirmatory. Future studies should include larger and more diverse cohorts, longer intervention periods, systemic biomarker analyses, prespecified endpoints, and direct performance measures to establish clinical relevance and reproducibility.

### 4.1. Limitations

Skin-gas measurements were exploratory, and the absence of blood biomarkers limits mechanistic interpretation and precludes using skin gas as a validated surrogate of systemic SCFA or ammonia exposure. First, the sample size was relatively small, which reduced the statistical power, particularly for subgroup and cluster analyses. Second, the intervention period was limited to 4 weeks, which may not be sufficient to capture stable microbiota adaptations; longer-term studies are warranted to confirm whether the observed changes persist. Third, the exclusive inclusion of male athletes limits generalizability; future trials should include female participants. Fourth, all participants consumed protein supplements, which may have influenced the gut environment independently of probiotic supplementation. Fifth, mechanistic interpretations were based on changes in metabolites and microbiota composition without direct functional or enzymatic measurement; the absence of metagenomic, metatranscriptomic, and host biomarker data limits causal inference. Sixth, exercise performance outcomes were not included, precluding direct evaluation of the link between metabolic changes and physical function. Finally, routine gut microbiota profiling is not currently feasible in most real‑world sports nutrition contexts, which limits the practical applicability of microbiota‑based stratification or personalized nutrition approaches.

Taken together, these limitations highlight the need for future confirmatory trials that prospectively prespecify primary endpoints, incorporate systemic biomarkers such as circulating SCFAs and nitrogen metabolites, consider longer intervention periods, recruit female athletes, and include exercise performance outcomes to strengthen causal interpretation and determine the functional relevance and generalizability of BB536‑related effects.

Importantly, no significant between‑group differences were observed in the full analysis population. The responder and enterotype subgroup analyses were conducted post hoc, were not prespecified, and involved small sample sizes, which increases the risk of type I error and may overestimate subgroup‑specific effects. The responder definition was outcome‑dependent (potential circularity), and enterotype clustering was exploratory rather than predetermined. Consequently, the subgroup‑specific findings should be interpreted as preliminary and hypothesis‑generating.

To further enhance analytical independence in future sponsor‑involved studies, involvement of an independent statistician will be considered. Because the probiotic capsules were manufactured under proprietary commercial specifications, certain manufacturing details cannot be disclosed. However, the viable count of the batch used in this study was measured and reported, and all products were manufactured under GMP‑compliant procedures.

## Supplementary Material

Supplementary Material Supplementary_Table_for_revise.xlsx

## Data Availability

Data supporting the findings of this study are available from the corresponding author upon reasonable request.

## Data integrity measures

To ensure data integrity and transparency despite sponsor involvement, multiple safeguards were implemented. First, the study was prospectively registered and approved by the institutional ethics committee. All study personnel, including investigators, data managers, and sponsor staff involved in study operations, remained blinded to group allocation until the clinical database was formally locked on July 25, 2025. The allocation list was maintained solely by an independent administrator, and code‑breaking was conducted only after database lock according to a predefined procedure.

All prespecified analyses conducted after unblinding were performed by sponsor-employed statisticians in strict accordance with the Statistical Analysis Plan (SAP). Exploratory post‑hoc analyses conducted by the sponsor after unblinding were independently reviewed and verified by academic investigators and were not used to modify or influence the results or interpretation of the prespecified analyses.

The sponsor had no access to unverified statistical outputs and did not influence the interpretation of the findings or the final conclusions of the study.
